# Role of Intermediate Filaments in Blood–Brain Barrier in Health and Disease

**DOI:** 10.3390/cells10061400

**Published:** 2021-06-05

**Authors:** Ece Bayir, Aylin Sendemir

**Affiliations:** 1 Ege University Central Research Test and Analysis Laboratory Application and Research Center (EGE-MATAL), Ege University, 35100 Izmir, Turkey; ece.bayir@ege.edu.tr; 2 Department of Bioengineering, Faculty of Engineering, Ege University, 35100 Izmir, Turkey; 3 Department of Biomedical Technologies, Graduate School of Natural and Applied Science, Ege University, 35100 Izmir, Turkey

**Keywords:** blood–brain barrier, vimentin, tight junctions, adherens junctions, endothelial permeability

## Abstract

The blood–brain barrier (BBB) is a highly selective cellular monolayer unique to the microvasculature of the central nervous system (CNS), and it mediates the communication of the CNS with the rest of the body by regulating the passage of molecules into the CNS microenvironment. Limitation of passage of substances through the BBB is mainly due to tight junctions (TJ) and adherens junctions (AJ) between brain microvascular endothelial cells. The importance of actin filaments and microtubules in establishing and maintaining TJs and AJs has been indicated; however, recent studies have shown that intermediate filaments are also important in the formation and function of cell–cell junctions. The most common intermediate filament protein in endothelial cells is vimentin. Vimentin plays a role in blood–brain barrier permeability in both cell–cell and cell–matrix interactions by affecting the actin and microtubule reorganization and by binding directly to VE-cadherin or integrin proteins. The BBB permeability increases due to the formation of stress fibers and the disruption of VE–cadherin interactions between two neighboring cells in various diseases, disrupting the fiber network of intermediate filament vimentin in different ways. Intermediate filaments may be long ignored key targets in regulation of BBB permeability in health and disease.

## 1. Introduction

Specialized endothelial cells covering the inner surfaces of the blood and lymph capillaries as a monolayer act as barriers, separating the blood or lymph fluid from the tissues [1]. The blood–brain barrier (BBB) is an endothelial cell-based, very specialized barrier system, which has great importance in providing brain homeostasis, regulating substance transport from blood to brain, and protecting the brain from pathogens and toxins [2]. The different cell types in the microenvironment of the endothelial cells, the basal lamina and the mechanical stimuli that cells are exposed to due to blood flow and vascular movement affect both the endothelial cell–matrix and the cell–cell interactions [3,4]. Therefore, these external stimuli cause the rearrangement of cytoskeleton proteins, controlling the structure of cellular junctions and the regulation of the endothelial barrier function. Although actin and microtubules are the cytoskeleton proteins whose role in the endothelial barrier function has been studied the most, the role of the intermediate filaments has been recognized only recently. The main purpose of this review is to underline that researchers should not ignore the role of this extensive nanofibrillar network that connects the plasma membrane with the nucleus, while investigating BBB function in health and disease.

## 2. Blood–Brain Barrier (BBB) Structure and Function

### 2.1. Brain Vascular Architecture

**Figure 1:**
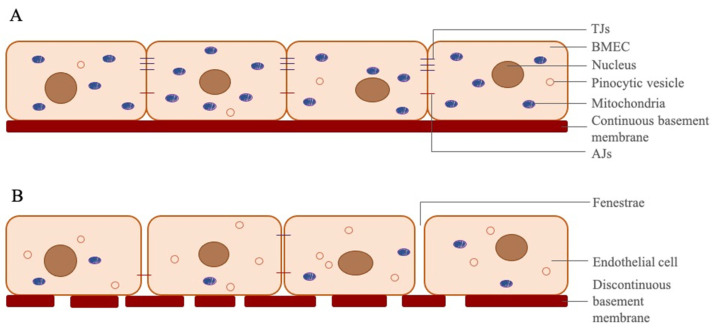
Differences between BMECs and peripheral endothelial cells. (**A**) BMECs line along a continuous basement membrane in cerebral capillaries. These cells have tight and adherens junctions to prevent paracellular transition of the molecules in bloodstream. BMECs have 5–6 times more mitochondria than peripheral endothelial cells, due to which transition of the molecules occurs mostly transcellularly in cerebral microvascular capillaries, and BMECs need more ATP than other endothelial cells. They have less pinocytic vesicles than peripheral endothelial cells to reduce transport of unwanted molecules via pinocytosis. (**B**) Peripheral endothelial cells, which have fenestrae, more pinocytic vesicles and less mitochondria, line along a discontinuous basement membrane in peripheral capillaries. These cells have fewer TJs and AJs, and sometimes have wide intracellular gaps to allow transition of molecules via diffusion.

The primary function of the circulatory system is to provide the nutrients and oxygen required for all tissues and organs of the body, and to remove cellular and metabolic wastes via the bloodstream. Since the central nervous system (CNS) is a specialized and critical system, in which vital functions are managed for the body, it requires an extremely stable microenvironment. For this reason, the structure and the barrier function of the circulatory system in the CNS also differs from that in the periphery, constructing the blood–brain barrier (BBB). BBB maintains ion homeostasis in the brain, prevents the brain from toxic and foreign substances and pathogens in the bloodstream [5,6,7]. The endothelial cells lining the peripheral blood vessels can have wide intercellular spaces, and their basement membranes are not continuous (Figure 1). While mass transport through the peripheral vessels mainly takes place by diffusion via intercellular space, BBB in brain micro vessels directs mass transfer to transcellular, paracellular and enzymatic pathways [8,9,10]. Brain microvascular endothelial cells (BMECs) reduce paracellular permeability by forming a large number of tight junctions (TJ), along with some adherens junctions (AJ) with adjacent BMECs. A continuous basement membrane also reduces transcytosis. Since BMECs have specific transporters, such as hexose transporters, amino acid transporters and monocarboxylic acid transporters, only molecules like glucose, glutamate and lactate that are recognized can pass transcellularly [11,12]. Some special cellular enzymes like acetylcholinesterase, alkaline phosphatase, γ -glutamyl transpeptidase and monoamine oxidases in BMECs were shown to inactivate some drugs, and neurotransmitters pass only transcellularly, which constitutes an enzymatic barrier [13]. Many previous studies on BBB permeability have focused on cell–cell junctions and paracellular permeability. However, to maintain the low permeability characteristic of the BBB, it has been shown that transcytosis must also be at a low rate, and the important effects of this mechanism on the BBB permeability has been demonstrated [14,15]. Ben-Zvi et al. have shown that the Mfds2a (major facilitator superfamily domain containing 2a) membrane protein expressed in CNS endothelium suppresses transcytosis [15]. The essential omega-3 fatty acid docosahexaenoic acid (DHA) transport is carried out by Mfds2a-mediated transport [16]. DHA prevents the formation of functional caveolae domains on the membrane. Thus, the formation of caveolae, which are invaginations of the plasma membrane, is prevented, and caveolae-mediated transcytosis is suppressed [14].

### 2.2. BBB Structure

**Figure 2:**
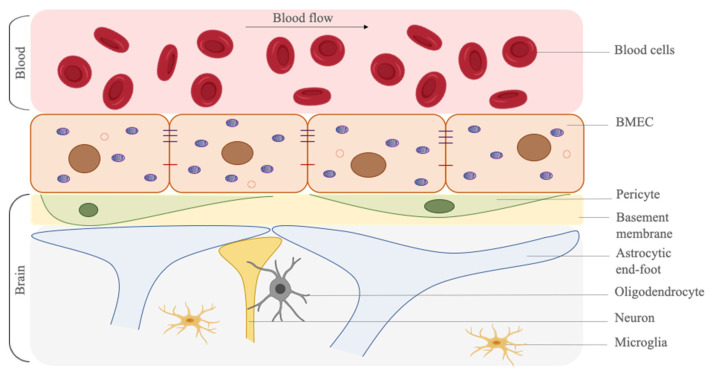
Schematic representation of the blood–brain barrier and the supportive cell types on the barrier function. BMECs form the lining of the brain microvasculature and they interact with multiple cells. Brain microvascular pericytes are embedded in the basal membrane and astrocytic end-feet surround brain micro vessels. Neurons, oligodendrocytes and microglia are also found in perivascular space. Created with BioRender.com (accessed on 21 May 2021).

BMECs are specialized cells that limit the movement of substances between the blood and the brain. However, there are also different cell types that support this structure and enhance the barrier function. These supporting cells are perivascular astrocytes, brain microvascular pericytes, oligodendrocytes, neurons and microglia. In addition to the cells, the basal membrane structure is also responsible for supporting the barrier structure and maintaining its functionality [17,18] (Figure 2). Perivascular astrocytes ensheathe the microvascular structure and support the integrity of the BBB. Brain microvascular pericytes directly control the endothelial cell behavior, as well as the vessel shape, by their contractile properties. Moreover, oligodendrocytes, neurons and microglia are also responsible for the BBB function; however, their mechanisms of action on BBB are not well-explained yet.

Endothelial cells are found in all vascular structures in the body and line the luminal surface of the vessel. Their main function is to regulate the transmission of molecules in the bloodstream to the surrounding tissues. BMECs are the most important component of the BBB barrier structure. They have many differences (e.g., fewer pinocytotic vesicles and fenestrae, more mitochondria and cell–cell junction proteins) from the peripheral endothelial cells [19,20,21]. All these differences ensure that paracellular and transcellular permeability from BMECs is lower than that of microvascular endothelial cells in the periphery.

BMECs are in constant communication with astrocytic end-feet and pericytes [22]. Pericytes, characterized by α-smooth muscle actin (α-sma), control the diameter of the capillaries by contracting and regulate the blood flow [2,23]. The area of pericytes covering the capillaries is inversely proportional to the capillary permeability [24]. Apart from regulating the capillary diameter and blood flow rate, pericytes also have crucial functions like controlling endothelial cell proliferation, regulating angiogenesis, secretion of extracellular matrix proteins and growth factors and regulating tight junction proteins [25,26].

Astrocytes cover almost the entire vascular surface with their cytoplasmic extensions, called end-foot [24,27]. Growth factors produced from astrocytes have an inducing effect on the barrier characteristics of BMECs [28]. Astrocytes contribute to the physical (by helping the formation of TJ’s), transcellular (by expression of specialized carrier proteins, e.g., P-glycoprotein) and enzymatic (by inducing of specialized enzyme systems, such as γ -glutamyl transpeptidase) barrier structures [29,30].

The basement membrane secreted by BMECs, astrocytes and pericytes is the extracellular matrix (ECM) that is mainly associated with cell surfaces [31]. It supports the cells with its layer-like structure of approximately 100 nm thickness [32]. It holds different cell groups together, regulates the signaling processes between different cells by associating with its ECM proteins (collagen IV, laminin, nidogen and perlecan) and regulates the barrier function by taking place between BMECs and astrocytes [17,31,33].

### 2.3. Tight and Adherens Junctions

**Figure 3:**
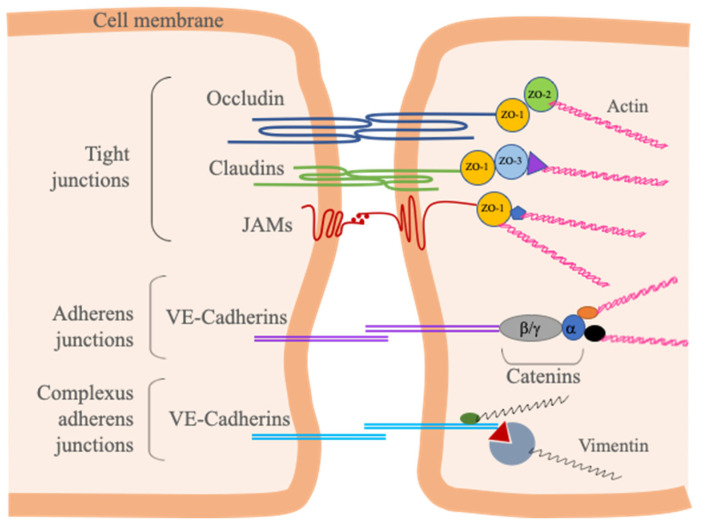
Major TJ and AJ proteins between adjacent BMECs. Transmembrane TJ proteins (Occludin, Claudins and JAMs) associate and bind to each other across the intercellular cleft. These proteins link to the F-actin cytoskeleton via zonula occludens proteins (ZO-1/2/3) and α-catenin. The most important component of BMEC AJs, VE-Cadherin, binds to the F-actin via catenins (α-catenin, β-catenin, γ-catenin, p120-catenin) and vimentin. VE-Cadherin is also the component of complexus adherens junctions, and links to vimentin by desmosomal plakoglobin/desmoplakin or p0071 linker proteins.

BMECs carry out the regulation of permeability through TJs and AJs. TJs located in the apical end of the lateral side of BMECs can even limit the passage of soluble substances by tightly connecting two neighboring cells like a zipper [34]. Proteins involved in the structure of TJs are divided into two, as transmembrane and cytoplasmic proteins. The transmembrane proteins occludin, claudins and junction adhesion proteins (JAMs) interact with cytoplasmic scaffolding proteins, which are Zonula Occludens 1–2 and 3 (ZO-1/2/3), members of membrane-associated guanylate kinase (MAGUK) protein family, actin cytoskeleton and associated proteins (Figure 3) [35,36,37].

AJs, which are not as tight as TJs, are adhesive structures; however, they also support TJs by connecting adjacent cells to each other [38]. Apart from initiating and stabilizing cell–cell adhesion, AJs have many other functions, such as regulating the actin cytoskeleton, providing cell signaling and transcriptional regulation [39,40]. If the structure of AJs that forms the connections between cadherin superfamily and actin bounded catenin is disrupted, the barrier structure is also disturbed. AJs and TJs have close functional interdependence [35,36,37].

Different than epithelial cells, endothelial cells do not have desmosomes that connect the intermediate filament network of adjacent epithelial cells. Instead, intermediate filaments are linked to AJs in endothelial cells through a complex called complexus adherens (Figure 3) [41]. These desmosome-like complexes link vimentin to VE-cadherin by desmosomal plakoglobin/desmoplakin or p0071 linker proteins [42,43]. Since desmosomes are known to provide junction stability and maintain tissue integrity in other cell types [44], in the lack of desmosomal junctions, it is plausible that AJs take over this duty by connecting intracellular intermediate filament networks through complexus adherens.

## 3. Endothelial Cytoskeleton

Endothelial cells line the vessel wall that contact with blood and protect the blood vessel and the surrounding tissue against mechanical stimuli originated from blood flow [45]. Besides maintaining the barrier function, these cells have some other roles, such as remaining to attach onto the matrix as a monolayer, maintaining a flat endothelial morphology to prevent turbulent flow, and covering the damaged area by proliferating and migrating rapidly when damage occurs in the vascular structure [46,47]. Endothelial cells need cytoskeletal proteins to perform all these tasks. Like other mammalian cells, the endothelial cell cytoskeleton consists of three filamentous proteins: actin microfilaments, microtubules and intermediate filaments [46,48,49]. These structures are in constant communication with each other directly or indirectly.

### 3.1. Actin Filaments

The actin protein is found in the endothelial cells in monomer form as globular actin (G-actin), or as filamentous actin (F-actin), which is the polymerized form (7 nm diameter) of G-actin monomers [50]. G- and F-actin are in balance in the cell, and these two types of actin protein react to cellular stimuli by rapidly polymerizing and depolymerizing with the help of actin-binding proteins and actin regulating proteins [51,52]. According to the ratio of G- and F-actin in the cytoskeleton, the cellular functions of actin are regulated. F-actin is involved in cell shape, polarity, cell–cell and cell–matrix interactions, cell migration and cellular transport mechanisms [53,54]. F-actins form membrane cytoskeleton, stress fibers and cortical actin ring (also called cortical actin rim) structures in endothelial cells [48].

**Figure 4:**
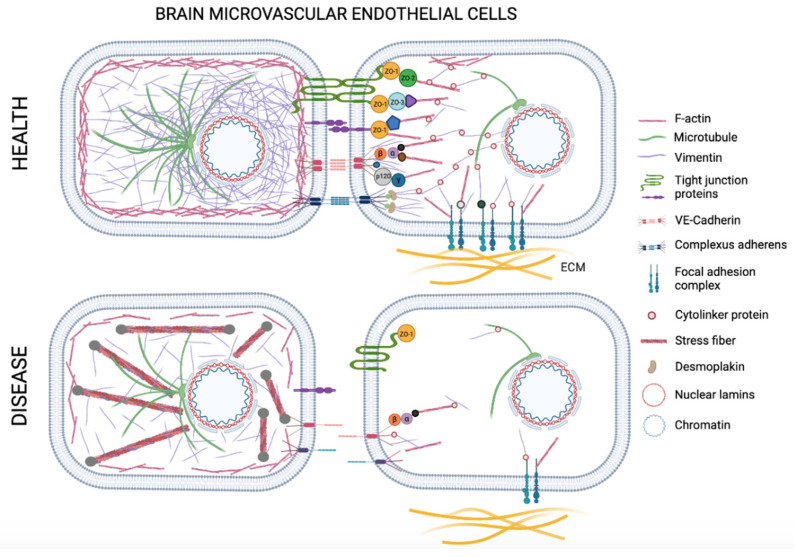
Schematic representation of the cytoskeleton and the interactions of transmembrane proteins with cytoskeleton proteins and the nucleus in brain microvascular endothelial cells (BMECs) in health and disease. In healthy conditions cortical actin ring supports and stabilizes the cell membrane outward. Cytoskeleton and cortical actin ring join cell–cell and cell–matrix adhesion complexes. Microtubules interact with actin directly or indirectly through intermediate filament proteins or signal molecules. Vimentin intermediate filament provides structural support and mechanical integrity to the cells. Transmembrane tight junction proteins, adherens junction and complexus adherens proteins associate and bind to each other across the intercellular cleft. These proteins link to the cell cytoskeleton directly or via cytolinker proteins. In disease conditions vimentin network is destroyed, the cell loses actin organization, and stress fibers increase in endothelial cells creating higher contractility. The adhesion complex structure reorganizes, and because of the loosening of cell–cell and cell–matrix junctions, gaps occur in the intercellular connections. Cells pull out from each other and barrier permeability increases. Created with BioRender.com (accessed on 21 May 2021).

Membrane cytoskeleton is a layer approximately 100 nm thick, attached to the cell membrane and is a separate structure from the cortical actin ring consisting of longer F-actin bundles [55]. However, these two structures are in communication with each other. The cortical actin ring structure provides a centrifugal force to the cell, supporting and stabilizing the cell membrane outward [55,56]. Thus, cell–cell and cell–matrix interactions are supported. Membrane cytoskeleton and cortical actin ring join TJ and AJ proteins and structures that form cell–matrix adhesion complexes, providing the formation and protection of the endothelial barrier (Figure 4). The first interaction between neighboring endothelial cells occurs with the help of lamellopodia, filopodia and junction-associated intermittent lamellipodia (JAIL) [53,57]. The space between two neighboring cells is reduced with the help of these cellular protrusions, and the first adhesion complexes are formed by the homophilic interaction between the extracellular amino termini of the VE-cadherins [53,58,59]. The carboxyl termini in the cytosolic part of the VE-cadherins are connected to the actin cytoskeleton via intracellular anchoring molecules, such as p120-catenin, α-catenin, β-catenin and γ-catenin, and actin-binding proteins, such as α-actinin and vinculin, and thus the two cells are interconnected [48,60,61]. There is a reorganization between F-actin that makes up the cortical actin ring and stress fibers. For example, during inflammation, stress fibers increase in endothelial cells with the increase in cytosolic Ca^2+^, decrease in cAMP, and activation of RhoA/Rho kinase pathway [48,62]. These stress fibers increase intracellular tension and with the reorganization of the adhesion complex structure, gaps occur in the intercellular connections [63]. Cells pull out from each other and barrier permeability increases. Therefore, the cortical actin ring is required for a linear and continuous AJ structure, hence a barrier structure with low permeability.

### 3.2. Microtubules

Microtubules are 25 nm-diameter fibers consisting of approximately 13 parallel protofilaments composed of alpha and beta-tubulin subunits [64,65,66]. Microtubules are located radially and densely in the center of the cell and their density decreases towards the cell membrane (Figure 4) [67]. Most of the microtubules are attached to the centrosomes; however, non-centrosomal microtubules are also found in the cytosol [68]. Microtubules are involved in many important processes, such as cell migration, spreading, division, polarization, cytoplasmic transport of signal molecules and vesicles and changing the shape of the cell [67,69]. Microtubules are in a constant state of reorganization by polymerizing and depolymerizing rapidly [48]. They interact with actin directly or indirectly through intermediate filament proteins or signal molecules. In the initial phase of endothelial barrier dysfunction, it has recently been observed that microtubules have a higher effect than actin filaments [70]. Depolymerization of microtubules occurs much faster than the reorganization of F-actin when a stress factor is applied to the cell. Even with minimal depolymerization of peripheral microtubules, increased permeability has been shown without actin reorganization or changes in cell morphology [71]. As a result of the depolymerization of microtubules, it has also been shown that the barrier function is impaired by causing stress fiber formation with RhoA activation [48,72].

### 3.3. Intermediate Filaments

Intermediate filaments (IFs) are the toughest of all three classes of cytoskeleton elements. They provide a template between the plasma membrane and the nucleus with an extensive surface area for cellular organelles and other plasma elements. They are mostly associated with desmosomes and focal adhesions on the plasma membrane, providing mechanical integrity [73]. The role of actin filaments and microtubules on cell–cell interactions and the permeability of the endothelial barrier has been investigated in detail; for a long time, only these structures among the cytoskeleton proteins were thought to be effective on the barrier function. It has been shown that intermediate filaments also have important functions in providing this regulation [74,75,76]. However, still, the role of intermediate filaments has not been studied thoroughly and their effects are not fully elucidated.

Intermediate filaments are tissue-specific, where they show different protein expressions depending on the tissue type or developmental stage. Many IFs were shown to be upregulated in regenerating tissues, and they were shown to have critical roles in the embryonic development [77].

The most abundant intermediate filament protein in microvascular endothelial cells is vimentin [78,79]. Vimentin is a type III intermediate filament protein and is expressed in cells of mesenchymal origin. The vimentin protein can be found in non-filamentous form in the cytoplasm, on the membrane and extracellular site of the cell [79,80,81]. Non-filamentous vimentin is involved in cell–cell interactions, immune activation, homeostasis, tissue repair and relationship with pathogens [79]. Filamentous vimentin is approximately 10 nm in diameter and provides structural support and mechanical integrity to the cells [82,83]. In addition to being responsible for maintaining the shape of the cell, it plays a major role in protecting the vascular cells and tissue against various mechanical factors, such as shear stress or contractile forces [84,85,86]. Lack of vimentin was shown to lead to decreased contractile properties and inhibits the differentiation of embryonic stem cells (ESCs) into endothelial phenotype in vitro. VIM −/− ESCs also showed altered cell–cell interactions and failed to form embryoid bodies [82,83]. Therefore, filamentous vimentin indirectly plays an important role in the formation and regulation of the actin fibers. Vimentins are stable structures that are bound to focal adhesions in cell–matrix interactions by cytoskeleton linker proteins (Figure 4). They increase the resistance of the cells against shear stress by reinforcing the attachment of cells to the matrix [76,87,88]. As vimentin filaments are stretched more, they become more resistant to further deformation due to hierarchical interactions of their coiled-coil subunits. This property is called “strain stiffening” or “strain hardening” [89]. The ability of filamentous vimentin to stretch beyond its original length also prompts its role as an important mechanosensor anchored to focal adhesions, activating the major mechanosensory molecule, focal adhesion kinase (FAK), deforming the nucleus, and transferring the outside mechanical stimuli to the nuclear lamins that are the main form of intermediate filaments within the nucleus [90]. Lamins re-organize the chromatin structure within the nucleus, therefore direct the intracellular transcriptional machinery according to the mechanical stimuli, which is also a phenomenon termed as “mechanoepigenetics” [91]. α-Catenin acts as the main mechanosensor protein in AJs that regulate the cadherin-specific mechanotransduction transmitting the external mechanical information to the nucleus through vimentin network [42,92].

An interesting study by Reinitz et al. has shown that while human umbilical vein endothelial cells (HUVECs) change their morphology and elongate in the direction of physiological shear stress, human brain microvascular endothelial cells (HBMECs) do not show any change in cell morphology or orientation under the same flow conditions [93]. The same group has also shown that HBMECS also resist elongation in response to curvature, as opposed to HUVECs [94]. Their observations suggest that brain microvascular endothelial cells show a unique property of the BBB, where these cells are resistant to actin modeling and elongation under shear and strain to minimize total length of cell–cell junctions. Unfortunately, they did not investigate the vimentin modelling characteristics. Another recent in vitro study that used traction force microscopy to evaluate structural changes in human brain endothelial cells on geometrically defined surfaces has shown that increased cellular traction levels (due to stress fiber generation) are accompanied by increased permeability, whereas when the cortical actin is stabilized, the permeability drops [95]. Since it is well documented that the cytoskeletal organization and cellular shape, as well as the cortical actin structure is particularly maintained by intermediate filaments and their linker proteins that bind to microtubules and microfilaments [77], the unique role of vimentin network for the integrity of BBB becomes more pronounced. Particularly the linker protein plectin has binding domains that can cross-link with all three types of cytoskeleton elements. The non-polar nature of intermediate filaments as opposed to polar microfilaments and microtubules gives them the ability to de-polymerize and re-polymerize allowing routine reassembly of their network [73]. It has been shown that actin filaments re-orient first in response to mechanical strain, followed by microtubules [96]. The slowest response of intermediate filaments might also be pointing out to the stabilization of the whole cytoskeletal adaptation mechanism by intermediate filaments.

Quinlan et al. propose the existence of a separate desmosome–intermediate filament network in epithelial cells that is circumferential and that explains the mechanosensor role of intermediate filaments transducing external stimuli to nucleus through linker of nucleoskeleton and cytoskeleton (LINC) complex [97]. Since endothelial cells lack desmosomes, AJs replace the main mechanosensor role in endothelial cells. Interaction of cadherins with actin through α-catenin is the main actor for sensing of the external mechanical force, while presence of vimentin improves stability and transduction [42].

## 4. Potential Roles of Intermediate Filaments on BBB Permeability in Disease

It has been shown that, as a result of many pathological conditions (e.g., genetic factors, trauma, infection, neurodegenerative diseases, brain tumor, ischemic/hemorrhagic shock, environmental toxins) junctions between adjacent endothelial cells are affected, and BBB dysfunction develops [98,99]. Specific stressors, such as cytokines (e.g., TNF-α, IL-6) and chemokines (e.g., CCL3, CXCL12) released from damaged cells, free radicals and hypoxic conditions that may occur as a result of ischemia, anemia or brain tumors cause the formation of stress fibers by actin reorganization in endothelial cells [100,101,102,103,104]. With the increased acto-myosin activity in the cell, the cytoskeleton tension increases, and the cells are pulled away from each other as a result of contraction; thus hyperpermeability increases [98]. Many studies have shown that BBB permeability changes as a result of actin reorganization [105,106,107,108,109,110]. It is now known that actin reorganization can occur from disturbances in the organization of the intermediate filament network, as well as directly from structural changes of F-actins and microtubules [111]. In order to create internal tension in the cell, an acto-myosin system and intermediate filament network are required [76]. Besides, in a cell that lacks a healthy intermediate filament network, stress fibers begin to deform with F-actin reorganization because intracellular tension is not sufficiently provided, and a discontinuous AJ structure and BBB dysfunction emerge because cortical actin structure is disrupted [112].

It has been shown that a continuous AJ structure is provided by the binding of the actin-bound VE-cadherins to the network structure of mechanically stable cage-like vimentins via plectins [76]. Gregor et al. indicated that in fibroblasts that do not have plectin cytoskeleton linker protein that binds intermediate filaments to actin, microtubules, organelles and focal adhesions, the intermediate filament network was disrupted and therefore the mechanosensory mechanism in the cell was attenuated [112]. Plectin deficiency was also linked to disruption of AJs and TJs, as well as increased contractility [76]. These studies show that linker proteins are essential for many functions of intermediate filaments that allow the cells respond and adapt to stress. When the intermediate filament network is destroyed, the cell loses tensegrity or actin organization, which will have significant effects on tumor formation, hypertension or delayed wound healing, as well as reduction of BBB integrity. Vimentin is also often related with motility in several cell types, because of its control over cellular contractility. Recently, vimentin was associated with epithelial–mesenchymal transition (EMT), and metastasis in cancer [113,114]. Since cellular motility is not expected in preservation of BBB integrity, stabilization of the vimentin and the whole cytoskeletal network by the linker proteins is necessary in order to avoid barrier disruption.

Changes in cell–cell adhesion are associated with the phosphorylation of adhesion complex members [115]. Vimentin filaments reorganize by phosphorylation, which is a transient posttranslational modification [116,117]. Polymerization and depolymerization of vimentin are essential processes for cell cycle, cell migration, cell spreading and cell signaling. For example, the phosphorylation of vimentin is required to separate cells from each other during the cytokinesis [118]. However, as a result of the disassembly of vimentin, the junctions between cells related to VE-cadherin are weakened (Figure 4) [119]. Due to activation of various protein kinases, such as Protein Kinase A (PKA), Protein Kinase C (PKC), RhoA Kinase (ROCK), the head domain of vimentin is phosphorylated and depolymerization is triggered [120,121,122]. PKC is activated by inflammation mediators, such as bradykinin platelet-activating factor and thrombin, and phorbol esters, leading to disruption in the BBB [119,123,124].

Shear stress induced by fluid flow causes vimentin network to redistribute around the nucleus and the periphery near cell junctions. Tensegrity model explains the distribution of contractile forces and dissipation of energy by intermediate filament structure and the related cross-linking proteins, such as plectins, that connect intermediate filaments with microtubules and microfilaments [125]. Low levels of shear stress have been shown to have protective effects on BBB. It was shown that cerebral endothelial cells form tight junctions and improved barrier function in vitro under shear stress [126], whereas disturbed fluid flow causes BBB breakdown [127]. Our lab’s own experience also has shown that brain vascular endothelial cells show higher levels of TJ (ZO-1 and Claudin-5) and AJ (VE-Cadherin) mRNA expressions under physiological levels of flow induced shear, while there is significant decrease in expression levels of these mRNAs when the flow conditions simulate hypertension (Data not published). Besides hypertension, disturbed fluid flow can be associated to many different pathological conditions including dementia, Alzheimer’s, epilepsy and ischemia [127].

Besides flow induced shear, other forms of mechanical stress also alter BBB permeability. Exposure to repeated low-level blast overpressure, that is commonly experienced in athletes and military personnel was shown to disrupt BBB in a mouse model [128].

In a study showing the role of intermediate filaments in endothelial permeability, it was determined that the organization of the intermediate filaments was disrupted by a drug called Withaferin A (WFA) that is known to cause phosphorylation of vimentin; there was no noticeable change in actin distribution [75]. However, the fact that there was an increase in BBB permeability shows that the phosphorylation of vimentin directly affects the barrier function. In the same study, it was shown that the junctions between endothelial cells were not loosened by the inhibition of phosphorylation of vimentin [75]. Although the cell–cell junctions are not damaged, the increase in BBB permeability may indicate that vimentin influences transcellular permeability as well as paracellular permeability. Apart from that, vimentin affects the expression and organization of surface molecules that are critical for adhesion [129].

In addition to being involved in cell–cell junctions, vimentin is also a component of focal adhesion complexes that provide cell–matrix interactions (Figure 4) [130,131]. In the absence of vimentin, spatial organization of focal adhesions are altered, the resistance of endothelial cells to shear stress decreases and cell–matrix adhesion deteriorates [76,87,88].

It is known that hypoxic conditions, such as in injury, amyotrophic lateral sclerosis (ALS), carbon monoxide poisoning, respiratory arrest, low blood pressure or stroke, change the actin cytoskeleton structure by activation of mitogen-activated protein kinase (MAPK) and Rho Kinase signaling pathways in endothelial cells [74]. Vimentin phosphorylation occurs in rat pulmonary microvascular endothelial cells (RPMECs) exposed to hypoxic conditions, and endothelial cell permeability increases if the intermediate filament network is disrupted [74]. It has been shown in RPMECs that intermediate filaments collapse around the nucleus, redistribute and polymerize in the cell periphery and create a stabilized continuous network in response to hypoxia. Hypoxia also increases the ratio of insoluble/soluble vimentin; vimentin de-phosphorylates, and hypoxia-induced heat shock protein (HSP27) stabilizes the intermediate filament network [74]. Similar vimentin re-organization in brain capillary endothelial cells was also reported [132]. This organization is comparable to the response of vimentin network against shear stress, where the cell–cell junctions are protected against the environmental stressor through establishment of a strong morphological belt in the cellular periphery.

Histamine is a neurotransmitter produced by histaminergic neurons, mast cells and microglia in the brain [133]. It has been shown that histamine is increased during CNS diseases, such as Parkinson’s Disease, schizophrenia, trauma, ischemia and sleep-wake disorders, and is accumulated in different amounts in various parts of the brain [134]. In the study of Shasby et al., histamine increased the phosphorylation of vimentin with the adhesion complexes of AJ, VE-cadherin, β- and γ-catenin; additionally, histamine disrupted the VE-cadherin-vimentin interaction, and therefore obstructed the AJ structure on human umbilical vein endothelial cells (HUVECs) [115]. Consequently, it is thought that cell–cell interactions that are disrupted as a result of the increased phosphorylation of vimentin and AJ complexes during diseases that cause histamine increase may cause BBB dysfunction.

Most of the studies investigating the effects of intermediate filaments on endothelial permeability have been done on intracellular filamentous vimentin. However, it is known that besides the filamentous vimentin in the brain microvascular endothelium, there is also non-filamentous vimentin on the cell surface [135]. Surface vimentin can facilitate internalization of virus and bacteria and infection of cells [81,136,137]. It was shown that the surface vimentin binds to the invasion protein (IbeA) of *Escherichia coli K1* and internalin family of surface protein (InlF) of *Listeria monocytogenes*, both bacteria that are associated with meningitis, indicating the important role of vimentin on invasion of pathogens to the CNS through BBB [137]. Huang et al., indicated that bacterial meningitis has three characteristic properties, NF-κB activation, pathogen invasion and polymorphonuclear neutrophil transmigration (PMNT) across the BBB. In their study, it is shown that vimentin, which is an NF-κB regulator, IbeA induced NF-κB activation, pathogen invasion and PMNT across the BBB, is reduced in vimentin −/− mice [138]. Vimentin deficient mice also showed resistance to *Streptococcus agalactiae* induced meningitis [139]. Paradoxically, depolymerization of the filamentous vimentin in the case of inflammation can loosen the intercellular junctions and increase BBB permeability, while the surface vimentin may also help infection. Enterovirus A71, which is the cause of foot and mouth disease and encephalitis, was also shown to increase BBB permeability, as well as vimentin expression [140]. It has been shown that in case of an inflammation, vimentins of both brain vascular endothelial cells and lymphocytes reorganize and adhere strongly, and they facilitate the trans-endothelial migration of lymphocytes through BBB [141].

## 5. Concluding Remarks

Although researchers do not regard the effects of intermediate filaments on cell–cell interactions as much as F-actin and microtubules, studies in this field actually show how effective intermediate filaments are in regulation and integrity of BBB. Intermediate filaments constitute a scaffolding within the cytoskeleton that determines the cellular organization and stabilizes the cell–cell junctions for maintaining the integrity of BBB. As many neurodegenerative diseases, as well as traumatic injuries and infections are associated with disturbed homeostasis and altered hemodynamics within the central nervous system, role of intermediate filament network and the linker proteins to maintain the cell and junction stability is highly pronounced. Particularly, the contribution of intermediate filaments as mechanosensors, and their role in the orchestrated organization of actin cortex and microtubules in reaction to blood flow induced shear must be studied in more detail in vitro and in vivo in order to shed light on their role in BBB functions in health and disease. Use of novel imaging techniques like electron cryotomography, traction force microscopy or Förster resonance energy transfer (FRET) might provide an opportunity to examine the signal transduction pathways in molecular level in the crosstalk of intermediate filaments with the cell–cell junctions and other cytoskeleton/nucleoskeleton molecules. Vimentin-targeted therapeutic strategies might play an important role for controlling and even ameliorating the central nervous system pathologies.
